# Examining interrater agreement between self-report and proxy-report responses for the quality of life-aged care consumers (QOL-ACC) instrument

**DOI:** 10.1186/s41687-024-00705-z

**Published:** 2024-03-04

**Authors:** Claire Hutchinson, Jyoti Khadka, Matthew Crocker, Kiri Lay, Rachel Milte, David GT Whitehirst, Lidia Engel, Julie Ratcliffe

**Affiliations:** 1 Caring Futures Institute, College of Nursing and Health Science, Flinders University, Adelaide, South Australia Australia; 2 ROSA, Adelaide, South Australia Australia; 3 Faculty of Health Sciences, Simon Fraser University, British Columbia, Burnaby, Canada; 4 School of Public Health and Preventive Medicine, Monash University Health Economics Group, Monash University, Melbourne, VIC Australia

**Keywords:** Long-term care, Family members, Older adults, Proxy assessment, Quality of life, Quality indicators, Residential aged care, QOL-ACC

## Abstract

**Background:**

Quality of life is an important quality indicator for health and aged care sectors. However, self-reporting of quality of life is not always possible given the relatively high prevalence of cognitive impairment amongst older people, hence proxy reporting is often utilised as the default option. Internationally, there is little evidence on the impact of proxy perspective on interrater agreement between self and proxy report.

**Objectives:**

To assess the impacts of (i) cognition level and (ii) proxy perspective on interrater agreement using a utility instrument, the Quality of Life-Aged Care Consumers (QOL-ACC).

**Methods:**

A cross-sectional study was undertaken with aged care residents and family member proxies. Residents completed the self-report QOL-ACC, while proxies completed two proxy versions: proxy-proxy perspective (their own opinion), and proxy-person perspective (how they believe the resident would respond). Interrater agreement was assessed using quadratic weighted kappas for dimension-level data and concordance correlation coefficients and Bland-Altman plots for utility scores.

**Results:**

Sixty-three residents (22, no cognitive impairment; 41, mild-to-moderate cognitive impairment) and proxies participated. In the full sample and in the mild-to-moderate impairment group, the mean self-reported QOL-ACC utility score was significantly higher than the means reported by proxies, regardless of perspective (*p* < 0.01). Agreement with self-reported QOL-ACC utility scores was higher when proxies adopted a proxy-person perspective.

**Conclusion:**

Regardless of cognition level and proxy perspective, proxies tend to rate quality of life lower than residents. Further research is needed to explore the impact of such divergences for quality assessment and economic evaluation in aged care.

## Introduction

Quality of life forms the most important quality indicator for aged care [1]. In 2019, Australia’s aged care system was placed under a global negative spotlight as a consequence of a Royal Commission into Aged Care Quality and Safety that found incidences of severe abuse and neglect [2]. The COVID-19 pandemic further exposed the cracks in the aged care system; a situation that was mirrored in other countries, including the United Kingdom (UK) and Canada [3]. In response to the proceedings and final report of the Royal Commission, Australia’s aged care system is currently undergoing a series of reforms. Amongst these is an expansion of the National Quality Indicators Program to include two new older-person-centred and non-clinical measures of care quality for the first time: quality of life and quality of care experience [4]. These new quality indicators will be used alongside more traditional indicators of care quality (e.g., pressure injuries, falls and malnutrition) to provide information about the quality of residential care facilities to older people and their families, supporting consumer choice. The quality indicators will be incorporated into a new star rating system for Australian residential care facilities, similar to those operating in the United States and several other countries [5, 6]. The Quality of Life-Aged Care Consumers (QOL-ACC) instrument, a newly developed quality of life instrument for quality assessment and economic evaluation in aged care, is the instrument that has been selected for the measurement of quality of life [4]. The QOL-ACC is currently being rolled out nationally across more than 2,700 residential care facilities.

The QOL-ACC was developed, tested, and validated from the ground up, using a mixed-methods approach with older people accessing aged care in home and residential care settings [7–13]. The QOL-ACC is the first quality of life instrument developed from its inception with older Australians and can be used for quality assessment in aged care. The instrument was also developed as a preference-based (or ‘utility’) instrument and can be used for economic evaluation. Further planned research will explore the suitability of the QOL-ACC with populations of older people in other care settings, including the Australian health system.

Self-reported quality of life is preferable to proxy-reported quality of life wherever possible [14]. However, striving for self-reported quality of life is challenging in populations of older people due to the relatively high prevalence of cognitive impairment and dementia in older age groups. This is particularly true for aged care residents in Australia; 54% of aged care residents have a diagnosis of dementia and 70–80% are estimated to have some form of cognitive impairment [15]. When older people are unable to self-complete, e.g., due to the presence of severe cognitive impairment or dementia, family members or aged care staff (where a family member is not available) may be asked to provide a proxy assessment of the person’s quality of life. To facilitate proxy completion, the QOL-ACC has proxy versions that adopt two different perspectives. Version 1 adopts the traditional proxy perspective used in a wide variety of quality-of-life instruments, where proxies are asked to complete the QOL-ACC based on their own perceptions of the person’s quality of life (the ‘proxy-proxy’ perspective). Version 2 asks proxies to complete the instrument based on how they think the resident would respond (the ‘proxy-person’ perspective).

A systematic review of studies using self-completed and proxy-completed preference-based quality of life instruments with older people identified that proxies tend to report lower quality of life than older people [16]. Several longitudinal studies have also shown that agreement between self-report and proxy-report declines as dementia progresses [17, 18]. The main reasons for this trend are unclear and may be influenced by factors relating to the person, their proxy, and the measure itself. Potential reasons include declining self-awareness in the individual over time as dementia advances [19], increasing difficulties for people with dementia in self-reporting their own quality of life using text-based instruments, and/or the potential adaptation of people living with dementia to their declining health status [20]. Whatever the reason for the disparity between self and proxy completion, this highlights the need for assessment of the interrater agreement between self and proxy report for the QOL-ACC, given that facilities with higher proportions of residents unable to self-report their own quality of life may ultimately report lower quality of life assessments relative to facilities with a lower proportion of residents that are able to self-report due to this reporting-bias rather than due to facility or provider-level factors.

Low levels of agreement between proxy-reported and self-reported quality of life have been identified across multiple contexts, including adults with schizophrenia and cancer patients [21]. Two previous studies identified in the systematic review conducted by Hutchinson and colleagues [16] found there was greater agreement between self-reported and proxy-reported health-related quality of life using the EQ-5D-3 L when proxies adopted the proxy-person perspective relative to the proxy-proxy perspective [22, 23]. Similar findings have been reported for the EQ-5D-5 L [24]. At the dimension level, agreement has been shown to be stronger for more observable dimensions of the EQ-5D-5 L (e.g., mobility) compared with those that are less observable (e.g., anxiety/depression) [24]. No study to date has assessed the impact of proxy perspective on agreement between proxy-reported and self-reported quality of life using the QOL-ACC instrument.

Therefore, this study sought to provide valuable data in the Australian context by identifying the equivalency of self and proxy report for the QOL-ACC and sought to identify if one proxy perspective was closer to self-report than the other. This study also sought to contribute to the limited data internationally on the impact of proxy perspective on interrater agreement whilst also considering the impact of cognitive impairment, given that cognitive impairment is highly prevenient in this population. Consequently, this study sought to examine the agreement of self and proxy report considering (i) residents’ cognition level (no cognitive impairment and mild-to-moderate cognitive impairment) and (ii) the proxy perspective (proxy-proxy and proxy-person) adopted.

## Methods and materials

### Participants

Older people (aged 65 years or older), permanently resident in aged care, able to communicate in English, and having the capacity to provide informed consent were eligible to participate. Study participants were recruited from 10 aged care facilities across metropolitan areas of Adelaide and rural South Australia. Providers identified people who, based on their most recent assessments, were likely to meet the cognitive threshold criteria. These people were provided with information sheets about the project. A list of residents who provided initial consent to participate was passed to the research team. Members of the research team then attended each facility and approached the listed residents to confirm their willingness to participate and to go through the formal consent process. Residential care facility managers identified suitable family member proxies once residents had consented to participate in the study. For family members to be eligible to be invited to participate, they had to be aged 18 years or older and visit the resident regularly (ideally, at least once per month).

### Materials

An interviewer-facilitated survey was designed for residents and consisted of the following elements. First, residents completed sociodemographic questions including age, gender, country of birth, highest level of educational attainment, and length of time resident in the aged care facility. Second, the interviewer administered the Mini Mental State Examination (MMSE) with the resident [25]. The MMSE, which has a scoring range from 0 to 30, was used to assess cognition level. In accordance with published guidelines [26], the following classifications were used: 27 to 30, no cognitive impairment; 10 to 26, mild-to-moderate cognitive impairment. Residents scoring less than 10 on the MMSE were considered to have severe cognitive impairment and were not eligible to participate due to a lack of capacity to consent.

Residents were then asked to complete the self-report version of the QOL-ACC. The QOL-ACC consists of six dimensions: mobility, pain management, emotional well-being, independence, social connections, and activities. Each item has five frequency-based response options, ranging from ‘all of the time’ to ‘none of the time’. The scoring procedure for the QOL-ACC comprises a value set (i.e., a set of utility weights), ranging from − 0.564 to 1.000, derived from a valuation study using a discrete choice experiment with survival duration approach [27]. The value set was developed with a large sample of older Australians receiving aged care services in home and residential care settings [13]. The scores are interpreted on a 0 to 1 scale, where 0 (zero) is dead and 1 is full quality of life; negative values reflect states worse than dead.

The survey for proxies was designed for online or telephone administration with a research team member and consisted of the following elements. First, a series of sociodemographic questions, including age, gender, country of birth, highest level of educational attainment, and frequency of phone contact and visits with the resident. Second, family proxies were asked to complete the two proxy versions of the QOL-ACC. The order of administration in all cases was the QOL-ACC proxy-proxy version, followed by the EQ-5D-5 L (data not reported here), then the QOL-ACC proxy-person version. The dimensions and response options for the proxy versions are the same as the self-report version, the only difference is the instructions to the respondent. For version 1 (proxy-proxy), instructions read “*For each question, please mark the ONE box that best describes your relative/friend’s quality of life TODAY*”; for version 2 (proxy-person version), the instructions were “*For each question, please mark the ONE box that your relative/friend would choose to best describe his/her quality of life TODAY*”. Proxies were requested to complete the survey on the same day as their family member, or as soon as possible thereafter.

### Analysis

Descriptive analysis was conducted on sociodemographic data from residents and proxies and summary statistics (mean, standard deviation, median, and 25th and 75th percentiles) were calculated for the QOL-ACC utility scores. The Friedman test was used to test for statistically significant differences in utility scores between the three ‘raters’ (i.e., self-report, proxy-report with the proxy-proxy perspective, and proxy-report with the proxy-person perspective); this analysis was conducted using the full sample and within the cognition subgroups. The Friedman test was selected over the Kruskal-Wallis test because the three ratings correspond to a related unit (i.e., the resident and the proxy). Where the null hypothesis was rejected, Wilcoxon signed-rank tests were used to explore pairwise comparisons between the three rater groups. The Mann-Whitney U test was used to test for statistically significant differences in QOL-ACC utility scores between the cognition subgroups.

Interrater agreement for utility scores was assessed using concordance correlation coefficients (CCC) [28, 29] and Bland-Altman plots [30, 31]. The CCC ranges from − 1 to + 1, with positive (negative) values reflecting the strength of agreement (disagreement). To aid interpretation of absolute values, the following classifications were used (these are the same classifications used for the interpretation of kappa values, described below): 0 = none; 0.01 ≤ 0.20 = poor, 0.21 to ≤ 0.40 = fair, 0.41 to ≤ 0.60 = moderate, 0.61 to ≤ 0.80 = good, and 0.81 to 1.00 = very good [31]. The Bland-Altman plots allow for further exploration of the relationship between pairwise ratings (i.e., self-report and proxy-proxy, and self-report and proxy-person), providing an illustration of the level of agreement between raters’ scores across the QOL-ACC scoring range. The Bland-Altman plot is a plot that shows the difference between pairwise ratings (y-axis) plotted against the respective mean of the ratings (x-axis). To aid interpretation, the plot includes lines representing the mean difference and the ‘limits of agreement’, calculated as the mean difference ± 1.96 standard deviations of the difference. The limits of agreement provide a simple means for agreement to be subjectively assessed, based on the width of the limits, the proportion of observations beyond the limits, and the location of ‘outlier’ observations across the scoring range of the instrument. An important consideration in the subjective interpretation of Bland-Altman plots when assessing preference-based instruments is the ‘funnelling’ of observations at the upper end of x-axis, where the difference between two ratings will approach zero as the average approaches 1.00.

For interrater agreement at the dimension level, the quadratic weighted kappa was used [32]. Kappa values can range from − 1 to + 1, with positive (negative) values reflecting the strength of agreement (disagreement). Interpretation of absolute values was as follows: 0 = none; 0.01 ≤ 0.20 = poor, 0.21 to ≤ 0.40 = fair, 0.41 to ≤ 0.60 = moderate, 0.61 to ≤ 0.80 = good, and 0.81 to 1.00 = very good [31]. Confidence intervals for kappa statistics were obtained through bootstrapping with 1000 replications [33]. All agreement analyses were conducted on the full sample and by cognition subgroup.

Analysis was conducted in R version 4.2.1 [34] and STATA version 15.1 [35]. For all significance tests, findings were interpreted using a statistical significance level of 0.01. Reporting of consensus for adult proxy followed the guidelines of Lapin and colleagues [36].

## Results

### Participant characteristics

Sixty-three older adults from 10 residential aged care homes participated in the study (22 with no cognitive impairment, 41 with mild-to-moderate cognitive impairment). An additional three residents expressed willingness to participate but did not meet the cognitive threshold. Sixty-three family members (one family member per resident) participated as proxies. Table 1 reports characteristics for the resident and proxy participants. 65% of the resident sample were female, and the mean age was 87.6 years. In the proxy cohort, 79% were female, and the mean age was 66.5 years. The most common proxy-to-resident relationships were son or daughter (46%), daughter- or son-in-law (22%), and spouse/partner (16%). Proxy completion was the same day as resident completion for 42 (66.7%) of the 63 dyads.

**Table 1:** Sociodemographic characteristics for all study participants (residents and proxies). Values are numbers (percentages) unless stated otherwise.^a^

	Residents (*n* = 63)	Proxies (*n* = 63)
Age		
Mean (standard deviation)	87.6 (8.0)	66.5 (10.6)
Median (25th, 75th percentiles)	88.0 (81.0, 93.0)	67.0 (59.0, 73.0)
Gender		
Female	41 (65.1)	50 (79.4)
Male	22 (34.9)	11 (17.5)
Non-binary	0 (0.0)	1 (1.6)
Education		
Primary school	14 (22.2)	2 (3.2)
Some secondary school	24 (38.1)	12 (19.1)
Completed secondary school	9 (14.3)	15 (23.8)
Tertiary (vocational or university)	16 (25.4)	33 (52.4)
Living in residential care		
< 12 months	16 (25.4)	-
1–3 years	20 (31.8)	-
> 3 years	23 (36.5)	-
Country of birth		
Australia	48 (76.2)	50 (79.4)
UK	10 (15.9)	8 (12.7)
Other	4 (6.4)	4 (6.4)
Location		
Metropolitan	10 (15.9)	-
Regional	53 (84.1)	-
Relationship to resident		
Daughter/Son	-	29 (46.0)
Daughter-/Son-in-law	-	14 (22.2)
Spouse/Partner	-	10 (15.9)
Other	-	10 (15.9)
Employment status		
Retired	-	34 (54.0)
Employed full time	-	11 (17.5)
Employed part time/casually	-	10 (15.9)
Other	-	7 (11.1)
Visits / Phone calls to resident^b^		
Daily	-	5 (7.9) / 11 (17.5)
Most days of the week	-	10 (15.9) / 7 (11.1)
Once a week	-	32 (50.8) / 24 (38.1)
Fortnightly to monthly	-	4 (6.4) / 5 (7.9)
Rarely/Never	-	11 (17.5) / 12 (19.1)
Unable^c^	-	- / 3 (4.8)

^a^ Numbers do not always sum to the total because of missing data

^b^ In the last six months

^c^ Resident unable to speak or does not like speaking over the phone

### Differences in QOL-ACC utility scores

All participants provided complete data for the QOL-ACC instrument. In the full sample and by cognition subgroup, family member proxies, regardless of the proxy perspective, reported lower QOL-ACC utility scores compared with residents and mean proxy-person utility scores were lower than mean proxy-proxy utility scores (Table 2). The Friedman test indicated a statistically significant difference between the three rater groups in the full sample (Q(2) = 141.79, *p* < 0.01) (Table 2). On post-hoc pairwise comparison using Wilcoxon signed-rank test, statistically significant differences were found between resident and proxy utility scores when adopting both the proxy-proxy perspective (Z = 3.44, *p* < 0.01) and the proxy-person perspective (Z = 4.23, *p* < 0.01) but not between proxy-proxy and proxy-person (Z = 1.67, *p* = 0.10). There were also statistically significant difference across the three rater groups in the no impairment (Q(2) = 49.95, *p* < 0.01) and mild-to-moderate impairment (Q(2) = 90.73, *p* < 0.01) subgroups (Table 2). In the mild-to-moderate impairment subgroup, there were statistically significant differences between resident and proxy utility scores for the proxy-proxy perspective (Z = 3.22, *p* < 0.01) and the proxy-person perspective (Z = 3.86, *p* < 0.01). There were no statistically significant differences between proxy-proxy and proxy-person utility scores in the no impairment subgroup (Z = 0.90, *p* = 3.7).

**Table 2:** Descriptive and inferential statistics for QOL-ACC utility scores, for the full sample and by cognition subgroup

	Full sample (*n* = 63)	No impairment (*n* = 22)	Mild-to-moderate impairment (*n* = 41)	Comparisons across cognition subgroups
Self-report				
mean (SD)	0.768 (0.23)	0.786 (0.24)	0.759 (0.23)	Z=-0.49, *p* = 0.62
median	0.823	0.846	0.822	
25th & 75th perc.	0.63, 0.93	0.78, 0.93	0.62, 0.94	
Proxy-proxy				
mean (SD)	0.666 (0.25)	0.715 (0.26)	0.640 (0.25)	Z=-1.39, *p* = 0.16
median	0.738	0.761	0.714	
25th & 75th perc.	0.50, 0.86	0.54, 0.93	0.47, 0.80	
Proxy-person				
mean (SD)	0.642 (0.27)	0.679 (0.26)	0.623 (0.28)	Z=-0.82, *p* = 0.41
median	0.747	0.786	0.691	
25th & 75th perc.	0.47, 0.84	0.44, 0.88	0.54, 0.80	
Comparisons across *raters*	Q(2) = 141.79; *p* < 0.01	Q(2) = 49.95; *p* < 0.01	Q(2) = 90.73; *p* < 0.01	

*p*, p value; *perc.*, percentiles; *Q(2)*, Friedman statistic; *SD*, standard deviation; *Z*, Z score for Mann-Whitney U test

Regarding comparisons across impairment groups, QOL-ACC utility scores were lower in the mild-to-moderate impairment group compared with the no impairment group for all three rater groups (i.e., self-report and both proxy perspectives). Within each rater group, none of the differences between the mild-to-moderate impairment subgroup and the no impairment subgroup were statistically significant (Table 2).

### Interrater agreement: QOL-ACC utility scores

**Fig. 1:**
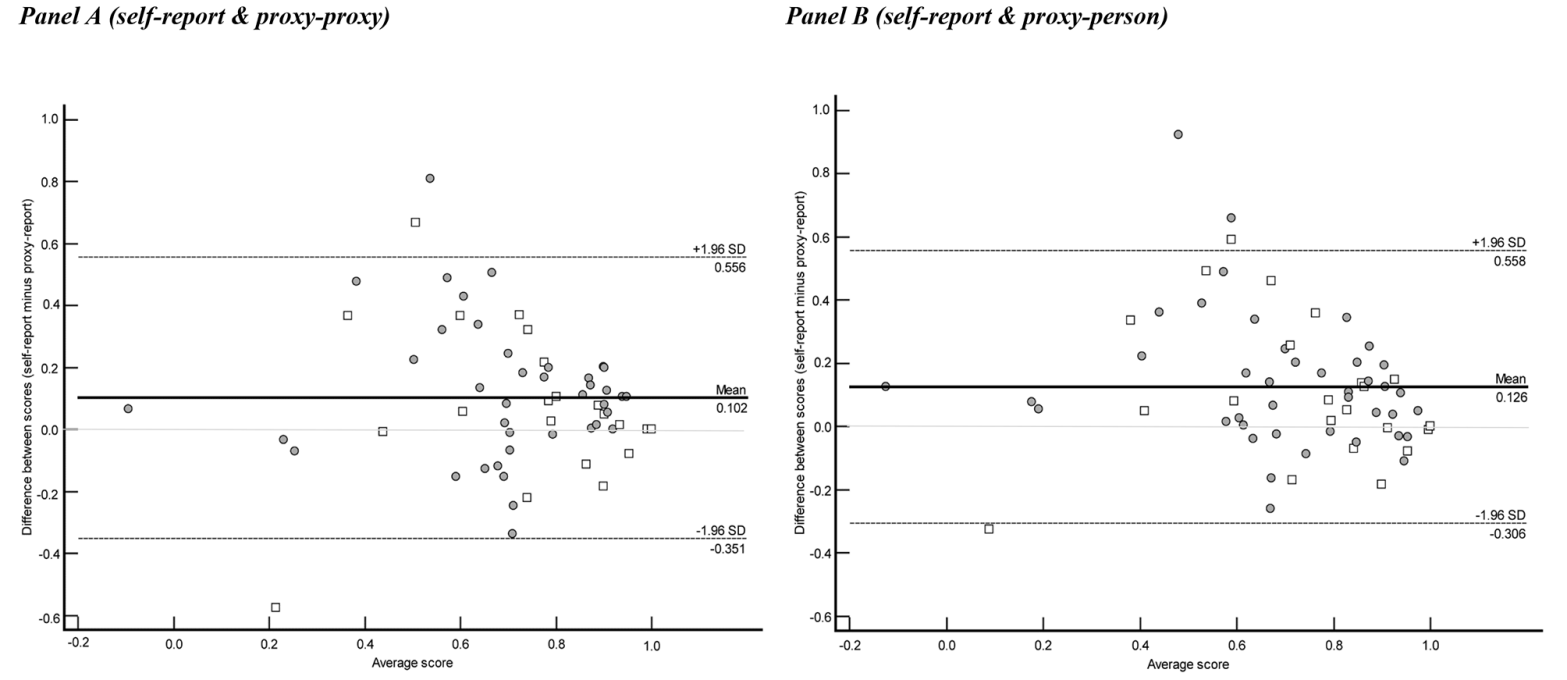
Bland-Altman plots showing the mean difference between self-report and proxy-report QOL-ACC utility scores (dark solid line) and associated 95% limits of agreement (dashed lines) when adopting the proxy-proxy perspective (panel A) and the proxy-person perspective (panel B). The markers plot the ‘difference between scores’ and the ‘average score’ for the respective analyses. For illustration, grey-fill circles identify the mild-to-moderate impairment subgroup and white-fill squares identify the no impairment subgroup. Panel A (self-report & proxy-proxy) Panel B (self-report & proxy-person)

Table 3 reports the concordance correlation coefficients for the full sample and by cognition subgroup. For the full sample, agreement was higher when proxies adopted a proxy-person perspective (0.557 compared with 0.507). Within cognition subgroups, the highest level of agreement was in the mild-to-moderate impairment group when a proxy-person perspective was adopted (0.563); the lowest level of agreement was for the no impairment group when proxies adopted the proxy-proxy perspective (0.466). Figure 1 shows the Bland-Altman plots, illustrating the relationships between QOL-ACC utility scores derived from self-report and proxy-proxy report (Fig. 1, Panel A) and self-report and proxy-person report (Fig. 1, Panel B). The wide limits of agreement (greater than 0.85 in both panels, which is more than 50% of the entire scoring range for the QOL-ACC) reflect the moderate levels of agreement reported in Table 3. The patterns of the differences further demonstrate the tendency for self-reported values to exceed proxy-reported values. For example, five of the six markers that lie outside the limits of agreement were when the difference was positive (proxy score < self-report score).

**Table 3:** Concordance correlation coefficients (95% confidence intervals) for QOL-ACC utility scores for the full sample and by cognition subgroup

	Full sample (*n* = 63)	No impairment (*n* = 22)	Mild-to-moderate impairment (*n* = 41)
Self-report & proxy-proxy	0.507 (0.33, 0.68)	0.466 (0.14, 0.80)	0.523 (0.32, 0.73)
Self-report & proxy-person	0.557 (0.40, 0.71)	0.537 (0.25, 0.82)	0.563 (0.39, 0.75)

### Interrater agreement: QOL-ACC dimensions

Table 4 reports agreement statistics for the QOL-ACC dimension-level comparisons. Of the 36 kappa statistics, 19 had a negative lower bound of the confidence interval (ranging between − 0.02 and − 0.37), interpreted as slight *dis*agreement. In contrast to QOL-ACC utility scores, agreement was generally found to be higher at dimension level when the proxy-proxy perspective was adopted. For the full sample, the highest level of agreement for both proxy perspectives was for the mobility dimension (proxy-proxy 0.375, ‘fair’; proxy-person 0.353, ‘fair’). The lowest level of agreement when adopting a proxy-person perspective was for activities (0.079, ‘slight’), whereas social connections was the lowest when adopting a proxy-proxy perspective (0.073, ‘slight’). Across the cognition subgroups, the highest level of agreement was for the activities dimension in the no impairment group when adopting the proxy-proxy perspective (0.422; ‘moderate’). For the proxy-proxy perspective, agreement on other dimensions in the no impairment group was either ‘fair’ (mobility, pain management, emotional well-being, independence) or ‘slight’ (social connections). For the proxy-person perspective, agreement in the no impairment group was ‘fair’ for four dimensions (mobility, emotional well-being, social connections, activities) and ‘slight’ for two (pain management, independence). In general, agreement was poorer between proxies and residents in the mild-to-moderate impairment group (an exception, for both proxy perspectives, being the mobility dimension).

**Table 4:** Agreement between proxy-reported and self-reported QOL-ACC dimension-level responses for the full sample and by cognition subgroup. Values are quadratic weighted kappa statistics (95% confidence intervals)

QOL-ACC dimension & rater pairing	Full sample (*n* = 63)	No impairment (*n* = 22)	Mild-to-moderate impairment (*n* = 41)
Mobility			
self-report/proxy-proxy	0.583 (0.32, 0.76)	0.522 (0.07, 0.78)	0.607 (0.25, 0.81)
self-report/proxy-person	0.544 (0.29, 0.74)	0.410 (0.177, 0.78)	0.603 (0.29, 0.81)
Pain management			
self-report/proxy-proxy	0.202 (-0.06, 0.47)	0.366 (0.12, 0.64)	0.092 (-0.23, 0.44)
self-report/proxy-person	0.078 (-0.19, 0.35)	0.215 (-0.03, 0.47)	-0.002 (-0.37, 0.41)
Emotional well-being			
self-report/proxy-proxy	0.288 (0.09, 0.48)	0.303 (0.05, 0.58)	0.280 (0.00, 0.52)
self-report/proxy-person	0.413 (0.17, 0.59)	0.389 (0.11, 0.65)	0.424 (0.10, 0.65)
Independence			
self-report/proxy-proxy	0.195 (-0.02, 0.48)	0.261 (-0.04, 0.62)	0.148 (-0.17, 0.46)
self-report/proxy-person	0.288 (0.05, 0.51)	0.321 (0.01, 0.67)	0.265 (-0.04, 0.60)
Social connections			
self-report/proxy-proxy	0.195 (-0.03, 0.464)	0.261 (-0.04, 0.63)	0.148 (-0.16, 0.49)
self-report/proxy-person	0.288 (0.05, 0.53)	0.321 (0.02, 0.66)	0.265 (-0.04, 0.62)
Activities			
self-report/proxy-proxy	0.144 (-0.08, 0.36)	0.351 (-0.08, 0.78)	0.072 (-0.16, 0.28)
self-report/proxy-person	0.068 (-0.14, 0.28)	0.232 (-0.19, 0.60)	0.003 (-0.25, 0.29)

## Discussion

This study examined the level of agreement between residents’ self-reported responses and family members’ proxy assessments using a quality of life instrument developed for aged care settings– the QOL-ACC. The study sought to assess the impact of residents’ cognition level and the adopted proxy perspective (proxy-proxy and proxy-person) on interrater agreement for QOL-ACC utility scores and QOL-ACC dimension-level responses. As has been widely observed in other studies comparing self-report and proxy report, for a range of quality-of-life instruments [16], residents rated their own quality of life higher than family member proxies, irrespective of the proxy perspective adopted. Significant differences in QOL-ACC utility scores were noted between residents and proxies, irrespective of the proxy perspective, such that self-report was higher.

No statistically significant differences were noted in self-reported quality of life across the cognition subgroups (within rater groups). This finding aligns with that of several other studies where cognitive subgroups were compared [37, 38], or where older adults with cognitive impairment were assessed longitudinally [39, 40]. The absence of association between cognitive impairment and self-reported quality of life has been argued to be because of lower levels of awareness over time as dementia progresses [19]. However, Clare [41] has argued that this is too simplistic as an explanation and that awareness is influenced by factors other than just cognition, including broader neuropsychological, psychiatric, and psychosocial explanations.

Notably, the QOL-ACC was developed with older adults including those with mild-to-moderate cognitive impairment. The development process included cognitive testing [42]. In the literature, cognition has been shown to impact on older adults’ ability to self-report as their cognitive impairment progresses [16]. However, a recently published paper on the use of think aloud protocols with aged care residents completing the QOL-ACC identified that residents could reliably self-report if their MMSE scores was 17 or above [43]. That is, they were able to interpret the items as expected by the developers and reflect on their own quality of life to make a response. In the current study, only five participants scored less than 17 on the MMSE, suggesting that the majority of this sample were likely able to reliably self-report. This evidence suggests that more residents should be able to self-report using the QOL-ACC than when using other quality of life instruments typically used with this population. For example, a think aloud study using the EQ-5D-5 L identified substantial issues in older adults understanding of the items and response categories [44] More research is needed on a larger sample to confirm these initial QOL-ACC findings to support providers choice and justification of self or proxy report for residents with cognitive impairment.

In terms of the impact of proxy perspective, for QOL-ACC utility scores concordance correlation coefficients were higher in the full sample and in the cognitive impairment subgroups when a proxy-person perspective was adopted. Such findings are important given that a significant proportion of older adults in residential care are not able to self-report their own quality of life [15]. These findings align with those of other research using the EQ-5D-3 L [22, 23] and EQ-5D-5 L [24]. However, at QOL-ACC dimension level, the opposite was found with agreement generally higher when the proxy-proxy perspective was adopted.

The QOL-ACC dimension-level analyses also highlighted the absence of consistent observations that can be drawn when comparing across cognition subgroups. Previous research, using EQ-5D instruments, has shown stronger agreement for physical or observable dimensions compared with psychosocial or non-observable dimensions [24, 45–47]. Our findings align with such evidence to some extent, with mobility having the strongest level of agreement in the full sample and the mild-to-moderate subgroup. The activities dimension may also be regarded as (relatively) observable, yet the kappa statistics were below 0.150 (‘slight’ agreement) in the full sample and mild-to-moderate subgroup. Given the preference-based nature of the QOL-ACC, agreement on items with larger utility weights (within the scoring algorithm) will have more impact on agreement at the utility score level. In this sample, mobility had the highest interrater agreement, and it is noteworthy that mobility is also the dimension with the largest utility weight in the QOL-ACC scoring algorithm.

When comparing proxy perspectives at the dimension level (Table 4), we observed that the kappa statistics were higher for the proxy-proxy perspective for the potentially more observable domains of mobility, pain management and activities, and higher for the proxy-person perspective for emotional well-being, independence, and social connections. However, these summary statements need to be interpreted with caution because the differences in kappa statistics when comparing proxy perspectives are often small.

The same preference-based scoring algorithm was applied to the QOL-ACC responses of all the raters. This algorithm was based on the preferences of a sample of older adults (*n* = 953) receiving aged care [13]. Therefore, the observed differences in QOL-ACC utility scores between residents and family proxies is attributable to the differences in the responses between residents and proxies to the QOL-ACC descriptive system.

Our findings concur with previous assertions that agreement in quality-of-life ratings between an older person and a family member proxy is likely to be multi-factorial and not solely influenced by the cognitive capacity of the older person [41]. Several studies have also demonstrated that within-proxy factors can impact the level of agreement in quality-of-life ratings, including the carers’ levels of anxiety and depression and their broader experiences that impact quality of life [46, 48]. Within-proxy factors could not be explored in the current study.

The findings from this study relating to the application of the QOL-ACC have potentially important implications for policy and practice including the roll out of the National Quality Indicators program in Australia, where they will be collecting self-report data and proxy data (e.g., from residents with severe dementia who are unable to self-report) on quality of life These findings indicate that asking proxies to adopt a proxy-person perspective is likely to be closer to self-report than adopting a proxy-proxy perspective, which is desirable if data is merged rather than treated as two separate sources of data, as this could impact on the subsequent generation of league tables in aged care.

These findings align with the emerging body of evidence also related to the EQ-5D-3 L and EQ-5D-5 L (widely applied in health and social care sectors internationally, an instrument which also has the two proxy versions available) indicating stronger agreement in overall quality-of-life ratings and corresponding utility scores when a proxy-person perspective is adopted [22–24]. This study adds to this emerging body of evidence that the perspective proxies are asked to adopt leads to different assessments of residents’ quality of life and which vary to differing degrees from older adults’ own assessments of their quality of life. These findings therefore has wider important potential relevance and applicability for quality assessment and economic evaluation in populations of older people across other sectors, including health systems, and in other countries.

### Limitations

This study has several limitations. The sample size comprised 63 residents and family member proxies, with residents recruited from 10 residential aged care facilities in one Australian state. Accordingly, caution is needed when generalizing the results. In particular, the participant group was under-representative of residents from culturally and linguistically diverse communities, who are estimated to make up ∼∼20% of aged care residents [49]. Due to resource limitations, only residents and proxies who could communicate in English were eligible to participate. In addition, proxy assessment was confined to family members only and hence residents for whom no family member proxy was available were unable to participate. Though 74.6% of proxies saw their resident family member at least weekly, 17.5% reported that they saw the resident ‘rarely/never’. Due to the sample size, it was not possible to conduct stratified analysis based on the frequency of contact. It would be helpful for future research to investigate the impact on inter-rater agreement of using alternative proxies, e.g., a close friend or aged care staff members. A further limitation was that although proxies were encouraged to complete their assessments on the same day as the residents, some were conducted at a later date (up to seven days later). As such, it is possible that the quality of life of a resident, as perceived by the respective proxy, may have changed during this time lag. Finally, the administration of proxy versions to family members was not randomised, with all family member proxies completing the proxy-proxy version before the proxy-person version, introducing the potential for question-order bias.

## Conclusion

Our study found lower QOL-ACC utility scores (self-report and proxies) and higher agreement between self-reported and proxy-reported QOL-ACC utility scores (for both proxy perspectives) in the cognitively impaired group when compared with the no cognitive impairment group. Albeit marginal, the agreement levels were higher between self-report and proxy-person than self-report and proxy-proxy, across both impairment subgroups, for QOL-ACC utility scores. This may indicate self-report and family member proxy-reported quality of life using the QOL-ACC instrument are influenced by the cognition level of the resident and the perspective adopted by proxies. Regardless of cognition level and proxy perspective, proxies tend to rate quality of life lower than aged care residents when using the QOL-ACC. Our study findings indicate that proxy-derived utility scores may more strongly align with residents’ self-assessment when the proxy-person perspective is adopted, although the differences in agreement across the proxy perspectives are small. At the dimension level, the picture is more mixed, with slightly higher levels of agreement for the proxy-proxy perspective on three of the six QOL-ACC dimensions. Further research in larger and more diverse samples of older people, and in other settings, is needed to better understand the generalizability of these findings and to assess the potential impact of divergences in self-report and family member proxy reported quality of life for quality assessment and economic evaluation in aged care.

## Data Availability

The datasets analysed during the current study are not publicly available due to the ethics agreement but are available from the corresponding author on reasonable request.
